# The Spanish Version of the Child Medical Fear Questionnaire: Cross-Cultural Adaptation and Validation

**DOI:** 10.3390/ijerph19010451

**Published:** 2021-12-31

**Authors:** Leticia San Martín-Rodríguez, Nelia Soto-Ruiz, Marta Ferraz-Torres, Cristina García-Vivar, Amaia Saralegui-Gainza, Paula Escalada-Hernández

**Affiliations:** 1 Department of Health Sciences, Public University of Navarre (UPNA), Avda. Barañain s/n, 31008 Pamplona, Navarra, Spain; leticia.sanmartin@unavarra.es (L.S.M.-R.); marta.ferraz@unavarra.es (M.F.-T.); cristina.garciavivar@unavarra.es (C.G.-V.); amaia.saralegui@gmail.com (A.S.-G.); paula.escalada@unavarra.es (P.E.-H.); 2 IdiSNA, Navarra Institute for Health Research, C/Irunlarrea, 3, 31008 Pamplona, Navarra, Spain; 3 Unit of Training and Research, Navarra Hospital Complex, C/Irunlarrea s/n, 31008 Pamplona, Navarra, Spain

**Keywords:** medical fear, children, psychometric properties, cross-cultural adaptation, Spanish

## Abstract

Having valid and reliable tools that help health professionals to assess fear in children undergoing medical procedures is essential to offer humanised and quality of care in the paediatric population. The aim of this study was to develop the cross-cultural adaptation and the evaluation of the psychometric properties of the Spanish version of the “Child Medical Fear Scale” in its shortened version (CMFS-R). The design consisted of two phases: first, of cross-cultural adaptation and second, of the psychometric validation of the CMFS-R with a sample of 262 children from Spain, applying a cross-sectional design. Confirmatory factor analysis was conducted to assess construct validity and the Cronbach’s alpha and the adjusted item-total score correlation coefficients were performed to study reliability. The results confirmed internal consistency and construct validity of the Spanish version of the CMFS-R, indicating that the scale has an acceptable level of validity and reliability. Therefore, this study brings a new version of the scale to assess fear related to medical procedures for use in the Spanish paediatric population.

## 1. Introduction

Most children are afraid of coming into contact with the healthcare environment, be it to see the doctor, go to the emergency room or hospital wards. Consequently, for the past few years, in the context of humanising healthcare, an attempt was made to make spaces visited by children look more attractive, featuring children’s themes, background music, toys and even healthcare professionals have begun to wear coloured uniforms to care for their younger patients [1,2].

The greatest fear for boys and girls visiting a doctor’s surgery or who have to go into hospital is being separated from their family, receiving injections for the different procedures and having to be in hospital for a long time [3,4,5].

Fear is an innate response that is regulated with maturity and that depends on each child. It is defined as a specific biological and psychological response to something real or imaginary [6]. Specifically, medical fear is defined as “fear of any experience that involves medical personnel or procedures involved in the process of evaluating or modifying health status in traditional healthcare settings” [7] (p. 10).

In 1988, Broome and colleagues [8,9] developed the “Child Medical Fear Scale” (CMFS) that, with 29 items, measured medical fear among children. The CMFS has demonstrated appropriate internal consistency, reliability, criterion and discriminant validity [3,10,11,12,13,14]. In relation to internal consistency reliability, the Cronbach’s α coefficient was greater than 0.70 in several studies, ranging between 0.93 and 0.78 [3,10,11,12,13,14].

Years later, the scale was reviewed and redundant items and items that showed little variability were eliminated, obtaining a 17-item scale (CMFS-R) [15]. The items included in the scale express statements of fear regarding certain aspects related to the health3care environment, which the child must answer according to a Likert-type scale with three points: not at all (0 points), a little (1 point) and a lot (2 points). So, the maximum level of fear is represented with a score of 34 points and the minimum level with zero points [11].

The discriminant validity of CMFS-R with pain measures was confirmed by Beyer and Knott [10] and Sparks [16]. Concurrent validity was established in r = 0.71; *p* < 0.05 and internal consistency, using Cronbach’s alpha, was also found to be adequate [10]. Regarding the construct validity, the original CMFS-R scale demonstrated that it had a 4-factor structure, that referred to medical procedural fears, healthcare environmental fears, intrapersonal fears (own bodies) and interpersonal fears (interaction with healthcare professionals) [11,17]. In their translation of the CMFS-R into Dutch, Abu-Saad et al. [18] determined the existence of two factors (environmental fears and procedural fears) that explained 30% of the variance, while in their translation into Thai, Chaiyawat and Brown [19] identified three factors (fear of physical hurt, fear of loss of control and interpersonal fears) that explained 42.15% of the variance.

The CMFS-R has been translated into three languages: Dutch [18], Thai and Chinese [11,19]. However, to date, no study has determined the psychometric properties of a Spanish version of the scale. Therefore, the aim of this study was to develop the cross-cultural adaptation and the evaluation of the psychometric properties of the Spanish version of the “Child Medical Fear Scale” in its shortened version.

## 2. Materials and Methods

This methodological study was performed in two stages: a first stage to translate the original instrument into Spanish and a second stage to analyse the psychometric properties of the Spanish version of the instrument, applying a cross-sectional design.

### 2.1. Phase I: Cross-Cultural Adaptation

Firstly, the statements for the instrument were translated and back-translated following the method proposed by Brislin [20]. Consequently, a native Spanish-speaker, who was familiar with the terminology being used, made an initial translation of the questionnaire into Spanish. An evaluation of the translation, to determine the equivalence of meaning for the items in the two questionnaires was carried out by four nurses who spoke the original questionnaire language, were also native Spanish-speakers and were familiar with the specific terminology in the questionnaire.

Secondly, to ensure linguistic validation [21], another bilingual person, independent from the first, who was a native English-speaker specializing in healthcare sciences performed the back translation from Spanish to English. Subsequently, a comparison was made between the original version of the questionnaire and the back translation, using a panel of experts composed of four nurse researchers with experience in cross-cultural adaptation of instruments and paediatric nurses. The panel discussed the differences they found and assessed the linguistic and conceptual equivalence and adaptation to Spanish culture. After discussion, consensus about the translation of all items was reached by the panel.

Thirdly, the questionnaire was piloted to verify the applicability of the instrument and the quality of the translation. To do this, a total of 5 children of different ages were selected to take the pretest on item comprehension. They were asked to indicate any items that might be confusing and to suggest an alternative when appropriate.

### 2.2. Phase II: Psychometric Validation

In a second stage, an evaluation was performed on the degree of validity and homogeneity of the questionnaire, by determining the content validity and the internal consistency of the statements.

#### 2.2.1. Sample

The sample included 262 children, aged between 6 and 10 years old, from 4 randomly selected schools in the city of Pamplona, in Spain. The random selection was done from the list of schools which had a collaboration agreement with the university to which the research team belongs, using the lottery method.

The children included in the sample had similar characteristics regarding their previous contact with the healthcare environment. The majority of them had experienced some of the common childhood illnesses and did not present serious pathologies.

#### 2.2.2. Data Collection

The data was collected between November 2019 and January 2020, using an online survey tool. An electronic questionnaire was created that included the 17 items in the CMFS-R questionnaire and two socio-demographic questions (age and gender). The children completed the questionnaire through a survey web tool (SurveyMonkey) using tablets in the classroom during class time. The students were given the necessary instructions to understand the task that was requested, and they were forbidden to speak during this time, which was monitored by the class teacher.

#### 2.2.3. Data Analysis 

Confirmatory factor analysis (CFA) was performed using LISREL 9.2 software. The aim was to use a series of structural linear equations to score the degree of adjustment for the data obtained via the Spanish version of the questionnaire, to the two known factor-based structures of the instrument: a 4-factor structure and another with 3 factors. Taking into account the ordinal nature of the data, the analysis was based on the polychoric correlation and asymptotic variance-covariance matrices [22,23,24,25]. The weighted least squares method was used to determine the model adjustment, as suggested by Jöreskog [24] when referring to ordinal data. The global fit of two conceptual structures to the data was evaluated using a set of indices [26]. The indices used were Reason for Fit (Pearson Chi-squared/degrees of freedom), RMSEA (Root Mean Square Error of Approximation), SRMR (Standardized Root Mean Square Residual), Tucker–Lewis index (TLI), Goodness-Of-Fit Index (GFI) and Comparative Fit Index (CFI) [26,27].

In addition, Cronbach’s α and the adjusted item-total score correlation coefficients were calculated to analyse internal consistency. These analyses were performed using SPSS 25.0 statistical software.

#### 2.2.4. Ethical Considerations

This study received approval from the Committee of Ethics, Animal Experimentation and Biosafety of the Public University of Navarre (PI-030/19). Written informed consent was obtained from the parents or legal guardians of the children. The collected data was anonymous since the data collected does not include the IP from which the survey was carried out. The confidentiality of the data was ensured by generating an identification code for each one of the participants and thus, no personal data were recorded.

## 3. Results

### 3.1. Phase I: Cross-Cultural Adaptation

As a result of the first stage of the study, a scale in Spanish that is equivalent to the original in English from a linguistic point of view was obtained (see Appendix A). 

After translation and back-translation of the items, the panel of experts identified one item that caused difficulty when interpreting its content. This item was “I am afraid of having my finger stuck”, whose literal translation in Spanish could result in different interpretations including trapping your finger in a door, having your finger “pinched” in a pulse oximeter, or having your finger stabbed to get capillary blood. After consulting the literature on the scale, it was decided to modify how the item was written to give it this latter meaning. 

Finally, the pilot test of the instrument showed that the understanding of one of the items by the participating children was adequate. Only one child noted that he did not know the meaning of the term “tongue blade”. The alternative “stick” (in Spanish: “palito”) was given for this term, thereby solving the comprehension problem. Taking this into account, it was decided to maintain the term “tongue blade” in the scale item, but that the child’s age would be considered, putting “stick” in brackets to clarify the term for younger children. 

### 3.2. Phase II: Psychometric Validation

The 262 children participating in this phase had a mean age of 8.27 years (Standard Deviation: 1.90) and 53.81% (*n* = 141) were boys. They came from four grant-maintained schools in the urban area of Pamplona, Spain. In these schools, girls and boys receive education in Spanish as a mother tongue. The statistical power offered by the 262 subjects is greater than 90%.

Firstly, the results from Barlett’s sphericity test (*p* < 0.001) and the Kaiser–Meyer–Olkin sample adequacy test (0.886) confirmed that the data were appropriate to carry out a factor-based analysis.

Table 1 presents the indices obtained from the CFA for the 4- and 3-factor structure that shows that the data adjust better to the 4-factor structure. However, according to the high correlation found among the 4 factors, it could be thought that there may exist a more general construct that underlies the 4 factors. Thus, a hierarchical CFA was performed by introducing one general factor as second-order factor. The results of the fit indices show a slightly worse fit for this model, therefore, the 4-factor structure was chosen as it was the most appropriate.

The items are distributed among the 4 factors as follows: Factor I, Intrapersonal, includes four items: I am afraid of (1) hurting myself, (4) seeing blood come out of me, (8) throwing up and (10) what I will say when I hurt. Factor II, Procedural, consists of five items: I am afraid of (2) going to the doctor’s office, (3) getting a shot, (6) having my finger stuck, (14) having the doctor or nurse look down my throat and (17) the doctor or nurse putting a tongue blade in my mouth. Factor III, Environmental, comprises four items: I am afraid of (5) going to the hospital, (11) if I went to the hospital, I would have to stay a long time, (13) I might die if I go to the hospital and (16) being away from my family if I go to the hospital. Factor IV, Interpersonal, incorporates four items: I am afraid that (7) the doctor and nurse will not tell me what they are going to do to me, (9) missing school if I’m sick, (12) my friends/family will catch something I have if I’m sick and play with them and (15) the nurse or doctor will tell me something is wrong with me [11].

**Figure 1:**
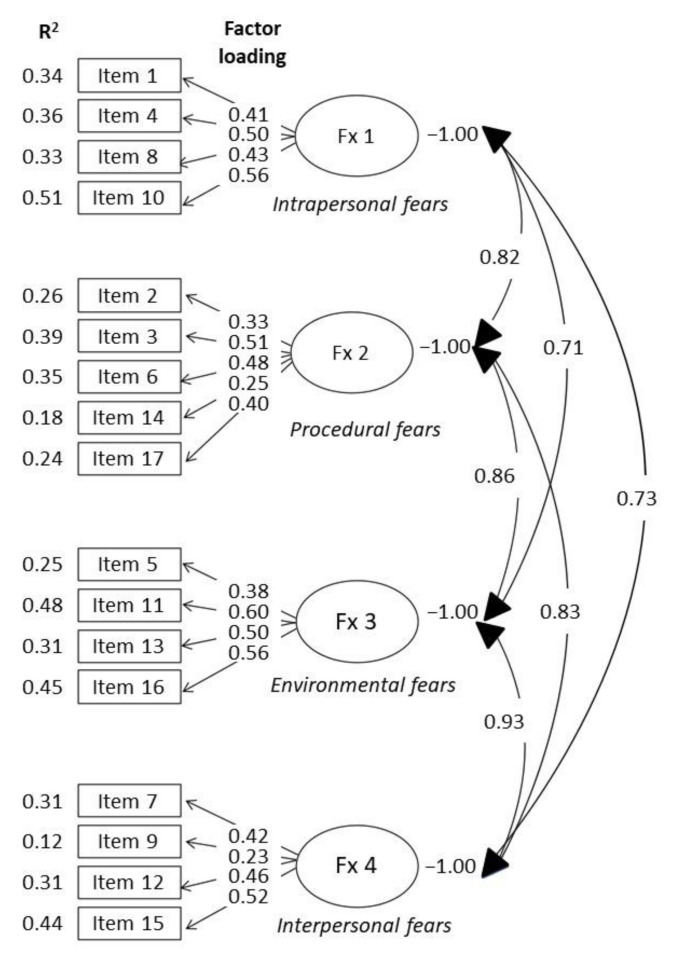
CFA for the 4-factor structure.

On the other hand, Figure 1 shows the estimated values for each of the parameters and the corresponding standard errors for each of the 17 items on the questionnaire, according to the 4-factor structure. In addition, the coefficients of determination (R^2^) for all 17 items vary in a range between 0.12 and 0.51.

Finally, the evaluation of the internal consistency of the instrument attained a Cronbach’s α of 0.87. The adjusted item-total score correlation coefficients varied between 0.355 and 0.612.

As it is presented in Table 2, the evaluation of the internal consistency of the 4 factors present Cronbach’s α coefficients between 0.60 and 0.70. In the same table, the Cronbach’s α coefficients with the item removed and the adjusted item-total score correlation coefficients are described.

## 4. Discussion 

The aim of this study was to develop the cross-cultural adaptation and the evaluation of the psychometric properties of the Spanish version of the CMFS-R, originally developed by Broome and Mobley [11], to assess children’s fear related to healthcare situations such as diagnostic or therapeutic procedures, hospitalization or surgery [12]. This research has evaluated both internal consistency and construct validity, in addition to its factorial structure through a confirmatory factor analysis. Our findings confirm that the Spanish version of the CMFS-R has been proven valid and reliable for its use in the Spanish context. The scale was easy to understand and took only around 7 min to complete.

The process of translating and back-translating the CMFS-R into Spanish was undertaken in accordance with established guidelines [20,21] to ensure content equivalence between the original and translated versions. The pilot process made it possible to ensure the applicability of the instrument and the adaptation of the translated terms that might generate the most difficulty for users to interpret, such as the item: “I am afraid of having my finger stuck”, ensuring that the translation is useful and valid. During data collection however, it was seen that the youngest children occasionally found it hard to understand the term “tongue blade” (“depresor” in Spanish). In this case, this was not due to translation issues, but due to lack of medical terminology knowledge among the younger participants. When the scale is used in the future, to ensure correct comprehension of this item, it is recommended to include a colloquial expression for tongue blade in brackets, such as stick (“palito” in Spanish). The difficulty with the medical terminology for school-age children has been observed in a range of studies [28,29].

At this point, it should be mentioned that the Spanish used for the scale translation is Spanish spoken in Spain. In other Spanish-speaking countries, particularly in Central America and South America, the Spanish features different expressions and terms, so to use it in these contexts, it would be necessary to adapt for language and culture.

The results of the confirmatory factor-based analysis have demonstrated that the Spanish version of the CMFS-R is a slightly better fit for the 4-factor structure proposed for the original questionnaire [11] (even with a 4-factor structure with a general factor), compared to the 3-factor structure in Chaiyawat’s study for the Thai translation [19]. Although Abu-Saad et al. [18] identified a 2-factor structure in the Dutch version of the CMSF-R, this structure was not included in the CFA because information about the items within each factor was not available. 

In relation to the fit of the model, the different indexes applied are within the values considered as good fit, except the GFI, which has a value of 0.89 (good fit >0.90). Delving into the literature, the GFI is considered an index highly influenced by the sample size [30,31], and there are even authors who propose to prioritise the CFI over the GFI [31]. On the other hand, it is also argued that in situations of discrepancy between SRMR (good fit) and the CFI or GFI (poor model fit) values, as it would be in the case of our study with the GFI value, it should be concluded that the model being tested provides a good fit [32].

Out of the four factors, Factor I, Intrapersonal, and Factor II, Procedural, encompass items related to unpleasant feelings such as pain or healthcare staff carrying out certain procedures. The items grouped into these two factors reflect the phobic agents classified in the blood-injection-injury phobias category listed by DSM-5 [33]. Factor III compiles factors referring to hospitalisation. The literature demonstrates that hospitalisation can be a particularly stressful experience for children due to their incomplete cognitive development, limited understanding of their disease or lack of strategies to deal with it [12,33,34]. Furthermore, the fear could increase as a consequence of being in an unknown environment [5]. Two of the items within Factor IV, Interpersonal, are related to the information provided by healthcare professionals. Several studies concluded that children wanted to know what was expected of them during consultations or procedures and what was going to happen to them then [35,36,37]. Even being with people they do not know generates fear [5].

Regarding the reliability of the instrument, the results show a good level of reliability. On the one hand, the Cronbach’s alpha located at 0.87, higher than the 0.70 set as satisfactory by Nunnally [38], indicates that 87% of the instrument variance is systematic; in other words, it represents the real differences between individuals regarding the perception of medical fear, while in 13%, it is due to random error [39,40]. The values of Cronbach’s α for each of the dimensions range between 0.60 and 0.70, three of them slightly below the reference value of 0.70. The lower number of items is an important limit to achieve a good level of internal consistency, as this is not based exclusively on the average correlation among items, but also on the number of items [38].

On the other hand, the adjusted item-total score correlation coefficients, also known as homogeneity indices or discrimination indices, and that represent the contribution of each statement to the whole instrument, put all of them above 0.30, a value set as satisfactory by some authors [41]. Therefore, the high correlation which has been found among items suggests that the concept has been measured with a high level of reliability in the sample. It is worth stressing that the reliability of an instrument may vary depending on the specific sample on which it is applied. The internal consistency reliability of the instrument in other languages has shown Cronbach’s α value between 0.93 and 0.66 [3,10,12,14], close to those found in our study. In particular, the Chinese translation of CMFS showed a Cronbach’s α value of 0.81 [17]. In the Thai version, the test-retest reliability coefficient was 0.80 [19]. Hence, the value for the Cronbach’s α coefficient obtained in this study indicates that the Spanish version of CMFS had an acceptable internal consistency.

As for reliability, it should be highlighted that this is not the property of an instrument but the property of an instrument administered to a specific sample, under specific conditions, in this case, a group of children between 6 and 10 years old from 4 schools in the north of Spain [40]. Consequently, it is important to continue using this Spanish version of the CMFS-R scale in other studies, with other samples, in other contexts, and evaluate its reliability in them. Additionally, in order to complete the psychometric evaluation of the Spanish version of the CMFS-R, future studies should analyse additional psychometric properties such as criterion validity or test-retest validity.

In their review, Foster and Park [12] identified potential disadvantages of the CMFS-R due to its limited psychometric support in terms of criterion, discriminant and construct validity and the insufficient testing performed with children from different cultural backgrounds. Our study helps compensate some of these limitations, as its validity has been proven in the Spanish culture. Furthermore, the results of our study offer additional evidence about the construct validity of this instrument.

The present study contributes to a better understanding of the medical fear experienced by children during medical visits or when performing procedures, which can have short- and long-term consequences on their physical and mental health [1,42,43]. It is related to patients’ negative results as a delay in recovery, increase in the pain level or greater likelihood of infection [12]. Given that it is such a frequent emotional response among the infant population, it is advisable that health professionals consider and handle it properly through healthcare for the paediatric population. Therefore, it is essential to find a convenient and evidence-based assessment tool to improve knowledge on this phenomenon. Using a reliable measuring instrument can also make it possible to evaluate the efficacy of the interventions designed to reduce the degree of medical fear in children. This study offers a valid and reliable instrument that can be used both in the clinical field and in research in the Spanish context, to score children’s fear in the medical environment.

## 5. Conclusions

This study presents a Spanish version of the CMFS-R scale, whose test in a “controlled” situation reports an acceptable level of validity and reliability. Further studies are required to measure validity in real clinical contexts to help reinforce the results obtained regarding the psychometric characteristics of the scale. The implementation of this scale and the use of a valid instrument to measure the fear experienced by the paediatric population with regard to the environment and the circumstances of the healthcare context is fundamental both in the clinical field and in research. This instrument will help to determine the most conditioning aspects that generate fear in paediatric patients, thus allowing developing interventions aimed at improving the negative experience that many children suffer when confronted with the healthcare context or in contact with physicians and nurses.

## Figures and Tables

**Table 1:** Indices.

Indices		Good Fit	3-Factor	4-Factor	1 × 4-Factor
Reason for fit	χ2/df	from 2 to 6	2.72	2.45	2.51
Root mean square error of aproximation	RMSEA	<0.08	0.081(0.070–0.093)	0.075 (0.063–0.086)	0.076 (0.065–0.087)
Standarized root mean square residual	SRMR	<0.08	0.065	0.064	0.064
Tucker–Lewis index	TLI	>0.90	0.92	0.94	0.94
Goodnes-of-fit index *	GFI	>0.90	0.88	0.89	0.88
Comparative fit index	CFI	>0.90	0.93	0.95	0.95

* GFI may be influenced by sample size.

**Table 2:** The evaluation of the internal consistency of the instrument.

	Cronbach’s α Coefficient	Cronbach’s α with the Item Removed	Corrected Item Total
** Factor I: Intrapersonal **	0.70		
1. I am afraid of hurting myself.		0.86	0.55
4. I am afraid of seeing blood come out of me.		0.86	0.47
8. I am afraid to throw up.		0.86	0.48
10. I am afraid of what I will say when I get hurt.		0.86	0.55
** Factor II: Procedural **	0.66		
2. I am afraid of going to the doctor’s office.		0.86	0.47
3. I am afraid of getting a shot.		0.86	0.52
6. I am afraid of having my finger stuck.		0.86	0.50
14. I am afraid of having the doctor or nurse look down my throat.		0.87	0.43
17. I am afraid of the doctor or nurse putting a tongue blade in my mouth.		0.87	0.43
** Factor III: Environmental **	0.69		
5. I am afraid of going to the hospital.		0.86	0.47
11. I am afraid if I went to the hospital, I would have to stay a long time.		0.86	0.61
13. I am afraid I might die if I go to the hospital.		0.86	0.48
16. I am afraid of being away from my family if I go to the hospital.		0.86	0.56
** Factor IV: Interpersonal **	0.60		
7. I am afraid the doctor and nurse will not tell me what they are going to do to me.		0.86	0.45
9. I am afraid of missing school if I’m sick		0.87	0.35
12. I am afraid my friends/family will catch something I have if I’m sick and play with them		0.86	0.50
15. I am afraid the nurse or doctor will tell me something is wrong with me.		0.86	0.57

## Data Availability

Due to ethical considerations, data are not available.

## Appendix A. “Child Medical Fear Scale” in Spanish

Instrucciones para los niños: Te voy a hacer unas preguntas sobre lo que piensas cuando estás enfermo, vas a un médico o vas al hospital. Quiero que me digas hasta qué punto te da miedo cada una de las frases que te voy a leer. Por ejemplo, si digo “Tengo miedo a vomitar si me pongo malo”, quiero que me digas si no tienes nada de miedo, si tienes un poco de miedo o mucho miedo de vomitar cuando estás malo. ¿De acuerdo? ¿Quieres preguntarme algo antes de empezar?

	**Nada De Miedo**	**Un Poco De Miedo**	**Mucho Miedo**
1. Tengo miedo a hacerme daño. 2. Tengo miedo a ir a la consulta del médico. 3. Tengo miedo a que me pinchen. 4. Tengo miedo a ver cómo me sale sangre. 5. Tengo miedo a ir al hospital. 6. Tengo miedo de que me pinchen en el dedo. 7. Tengo miedo a que los médicos y las enfermeras no me digan lo que me van a hacer. 8. Tengo miedo a vomitar. 9. Tengo miedo a no ir al colegio si me pongo malo. 10. Tengo miedo a llorar cuando me hagan daño. 11. Si voy al hospital, tengo miedo a tener que quedarme mucho tiempo allí. 12. Tengo miedo a contagiar a mi familia o amigos si estoy enfermo y juego con ellos. 13. Tengo miedo a morirme si voy al hospital. 14. Tengo miedo cuando el médico o la enfermera miran mi garganta. 15. Tengo miedo a que el médico o la enfermera me digan que me pasa algo malo. 16. Tengo miedo a estar lejos de mi familia si voy al hospital. 17. Tengo miedo cuando el médico o la enfermera apoyan el palito en mi lengua.			

## References

[1] Dalley J.S., McMurtry C.M. (2016). Teddy and I Get a Check-Up: A Pilot Educational Intervention Teaching Children Coping Strategies for Managing Procedure-Related Pain and Fear. Pain Res. Manag..

[2] McMurtry C.M., Riddell R.P., Taddio A., Racine N., Asmundson G.J.G., Noel M., Chambers C., Shah V. (2015). Far From “Just a Poke”. Clin. J. Pain.

[3] Hart D., Bossert E. (1994). Self-reported fears of hospitalized school-age children. J. Pediatr. Nurs..

[4] Salmela M., Salanterä S., Aronen E.T. (2010). Coping with hospital-related fears: Experiences of pre-school-aged children. J. Adv. Nurs..

[5] Salmela M., Aronen E.T., Salanterä S. (2011). The experience of hospital-related fears of 4- to 6-year-old children. Child Care Health Dev..

[6] Nicastro E.A., Whetsell M.V. (1999). Children’s fears. J. Pediatr. Nurs..

[7] Steward M.S., Steward D.S. (1981). Children’s conceptions of medical procedures. New Dir. Child Adolesc. Dev..

[8] Broome M.E., Hellier A.P. (1987). School-Age Childrens Fears of Medical Experiences. Issues Compr. Pediatr. Nurs..

[9] Broome M., Hellier A., Wilson T., Dale S., Glanville C., Waltz C.F., Strickland O.L. (1988). Measuring children’s fear of medical experiences. Measurement of Nursing Outcomes: Vol: Measuring Client Outcomes.

[10] Beyer J.E., Knott C.B. (1998). Construct validity estimation for the African-American and Hispanic versions of the Oucher Scale. J. Pediatr. Nurs..

[11] Broome M., Mobley T., Strickland O.L., Dilorio C. (2003). The child medical fears scale. Measurement of Nursing Outcomes Volume 2: Client Outcomes and Quality Care.

[12] Foster R.L., Park J.-H. (2012). An Integrative Review of Literature Examining Psychometric Properties of Instruments Measuring Anxiety or Fear in Hospitalized Children. Pain Manag. Nurs..

[13] Mahat G., Scoloveno M. (2003). Comparison of fears and coping strategies reported by Nepalese school-age children and their parents. J. Pediatr. Nurs..

[14] Wilson A.H., Yorker B. (1997). Fears of Medical Events Among School-Age Children with Emotional Disorders, Parents, and Health Care Providers. Issues Ment. Health Nurs..

[15] Broome M.E., Bates T.A., Lillis P.P., McGahee T.W. (1994). Children’s medical fears, coping behaviour patterns and pain perceptions during a lumbar puncture. Eur. J. Cancer Care.

[16] Sparks L. (2001). Taking the “Ouch” Out of Injections for Children. MCN Am. J. Matern. Nurs..

[17] Jin Y. (1997). Selected Factors Associated with Medical Fear among Hospitalized Chinese School Age Children. Master’s Thesis.

[18] Abu-Saad H., Pool H., Tulkens B. (1994). Further validity testing of the Abu-Saad Paediatric Pain Assessment Tool. J. Adv. Nurs..

[19] Chaiywat W., Brown J.K. (2000). Psychometric properties of the Thai versions of state-trait anxiety inventory for children and child medical fear scale. Res. Nurs. Health.

[20] Brislin R.W. (1970). Back-translation for cross-cultural research. J. Cross-Cult. Psychol..

[21] Sperber A.D. (2004). Translation and validation of study instruments for cross-cultural research. Gastroenterology.

[22] Hayduk L.A. (1987). Structural Equation Modeling with LISREL. Essentials and Advances.

[23] Jöreskog K.G. (2002). Structural Equation Modeling with Ordinal Variables Using LISREL. https://ssicentral.com/wp-content/uploads/2021/09/ordinal.pdf.

[24] Jöreskog K.G., Bollen K.A., Long J.S. (1993). Testing Structural Equation Model.

[25] Muthén B. (1984). A general structural equation model with dichotomous, ordered categorical, and continuous latent variable indicators. Psychometrika.

[26] Aroian K.J., Norris A.E., Munro B.H. (2005). Confirmatory Factor Analysis. Statistical Methods for Health Care Research.

[27] Ral A., Varela J., Abalo J., Lévy J., Lévy J., Varela J. (2006). El análisis factorial confirmatorio. Modelización con Estructuras de Covarianzas en Ciencias Sociales: Temas Esenciales, Avanzados y Aportaciones Especiales.

[28] Koller D. (2017). ‘Kids need to talk too’: Inclusive practices for children’s healthcare education and participation. J. Clin. Nurs..

[29] Lööf G., Andersson-Papadogiannakis N., Silén C. (2019). Children’s own perspectives demonstrate the need to improve paediatric perioperative care. Nurs. Open.

[30] Mulaik S.A., James L.R., Van Alstine J., Bennett N., Lind S., Stilwell C.D. (1989). Evaluation of goodness-of-fit indices for structural equation models. Psychol. Bull..

[31] Tanguma J. (2001). Effects of Sample Size on the Distribution of Selected Fit Indices: A Graphical Approach. Educ. Psychol. Meas..

[32] Browne M.W., MacCallum R.C., Kim C.-T., Andersen B.L., Glaser R. (2002). When fit indices and residuals are incompatible. Psychol. Methods.

[33] American Psychiatric Association (2013). Diagnostic and Statistical Manual of Mental Disorders (DSM-5 (R.)).

[34] Dela-Fuentes B., Quintana M., Rimbau J., Martínez-Mejías A., Úriz M.S., Rivera-Pérez C., Garolera M. (2018). Anxiety, hospital fears and conduct and behavioral alterations during pediatric hospitalization. Actas Esp. Psiquiatr..

[35] Bray L., Appleton V., Sharpe A. (2019). The information needs of children having clinical procedures in hospital: Will it hurt? Will I feel scared? What can I do to stay calm?. Child: Care, Health Dev..

[36] Bray L., Appleton V., Sharpe A. (2019). ‘If I knew what was going to happen, it wouldn’t worry me so much’: Children’s, parents’ and health professionals’ perspectives on information for children undergoing a procedure. J. Child Health Care.

[37] Wecher S. (2014). School-Age Children’s Perception of Stress in the Hospital: A Drawand Tell Story. https://digitalrepository.unm.edu/nurs_etds/20.

[38] Nunnally J. (1994). Psychometric Theory.

[39] Pedhazur E.J., Schmelkin L.P. (1991). Measurement, Design, and Analysis: An Integrated Approach.

[40] Polit D.F., Beck C.T. (2004). Nursing Research: Principles and Methods.

[41] Ebel R.L., Frisbie D.A. (1986). Essentials of Educational Measurement.

[42] Jaaniste T., Hayes B., von Baeyer C.L. (2007). Providing children with information about forthcoming medical procedures: A review and synthesis. Clin. Psychol. Sci. Pract..

[43] Taddio A., Ipp M., Thivakaran S., Jamal A., Parikh C., Smart S., Sovran J., Stephens D., Katz J. (2012). Survey of the prevalence of immunization non-compliance due to needle fears in children and adults. Vaccine.

